# Effects of Diazepam on Oxidative Stress, Apoptosis, and Vascular Reactivity in Human Umbilical Arteries Under Experimental Hyperhomocysteinemia

**DOI:** 10.1155/bmri/5105153

**Published:** 2026-08-13

**Authors:** Milica Gajić Bojić, Zvjezdana Ritan Mičić, Aneta Stojmenovski, Anđela Bojanić, Sanja Jovičić, Milka Matičić, Nebojša Mandić-Kovačević, Snežana Uletilović, Ljiljana Amidžić, Miroslav Savić, Ranko Škrbić

**Affiliations:** ^1^ Center for Biomedical Research, Faculty of Medicine, University of Banja Luka, Banja Luka, Republic of Srpska, Bosnia and Herzegovina, unibl.org; ^2^ Department of Pharmacology, Toxicology and Clinical pharmacology, Faculty of Medicine, University of Banja Luka, Banja Luka, Bosnia and Herzegovina, unibl.org; ^3^ Clinic for Gynecology and Obstetrics, University Clinical Center of the Republic of Srpska, Banja Luka, Bosnia and Herzegovina, kc-bl.com; ^4^ Department of Histology and Embryology, Faculty of Medicine, University of Banja Luka, Banja Luka, Bosnia and Herzegovina, unibl.org; ^5^ Department of Pharmacy, Faculty of Medicine, University of Banja Luka, Banja Luka, Bosnia and Herzegovina, unibl.org; ^6^ Department of Medical Biochemistry and Chemistry, Faculty of Medicine, University of Banja Luka, Banja Luka, Bosnia and Herzegovina, unibl.org; ^7^ Department of Biology of Cell and Human Genetics, Faculty of Medicine, University of Banja Luka, Banja Luka, Bosnia and Herzegovina, unibl.org; ^8^ Department of Pharmacology, Faculty of Pharmacy, University of Belgrade, Belgrade, Serbia, bg.ac.rs

**Keywords:** apoptosis, arterial wall histology, diazepam, human umbilical arteries, hyperhomocysteinemia, oxidative stress, preeclampsia

## Abstract

In obstetrics, diazepam is commonly used to treat anxiety, insomnia and as a second‐line treatment for seizures in preeclampsia and eclampsia. However, the effects of diazepam on the oxidative balance and vascular function of the umbilical circulation are still insufficiently studied. In this study, we investigated the effects of suprapharmacological concentrations of diazepam in an in vitro model of human umbilical artery (HUA) injury, induced by incubation with 1 mM homocysteine, representing experimental conditions that mimic hyperhomocysteinemia. HUAs were divided into four groups: control (C), diazepam (D) alone (100 *μ*mol/L), homocysteine (Hcy) alone (1 mM), and combined treatment (HcyD). For oxidative stress assays, data were obtained from 8–10 individual HUA samples per group, with lipid peroxidation, nitrite (NO_2_^−^) levels, and SOD, CAT, and GSH activities determined spectrophotometrically. Histological and TUNEL analyses were performed in triplicate (*n* = 3 per group), whereas vascular reactivity experiments, using an organ bath system, included six individual vessel samples per group. Statistical analysis was performed using the paired Student′s *t*‐test (*p* < 0.05). Diazepam alone significantly reduced superoxide dismutase (SOD) activity, indicating an apparent impairment of antioxidant defenses. In contrast, concomitant treatment under hyperhomocysteinemia lowered the levels of thiobarbituric acid reactive substances (TBARSs) and NO_2_^−^, suggesting partial protection against lipid peroxidation and nitrosative stress. Histologically, diazepam preserved arterial structure with minor subendothelial changes, whereas cotreatment reduced homocysteine‐induced endothelial disruption, smooth muscle disarray, and medial connective tissue loss. Apoptotic index was significantly reduced with cotreatment comparing to hyperhomocysteinemia, whereas diazepam alone showed values similar to control. Functionally, diazepam significantly reduced serotonin‐induced contraction of HUAs, but this vascular effect was attenuated under hyperhomocysteinemic conditions. Overall, concomitant treatment with diazepam partially attenuated oxidative and structural changes induced by hyperhomocysteinemia in our experimental setting, in a preclinical model mimicking selected pathophysiological aspects of preeclampsia.

## 1. Introduction

Diazepam, a long‐established benzodiazepine, was historically reported as commonly prescribed in pregnancy based on older data (up to 20% in the third trimester), but more recent evidence suggests overall benzodiazepine use is closer to 1.9% (95% CI 1.6–2.2%), with notable regional variability and higher rates in Eastern Europe [1–3].

In addition to its clinical indications for anxiety and insomnia, diazepam is recommended as a second‐line treatment for the prevention of seizures in preeclampsia and eclampsia when magnesium sulfate is contraindicated or unavailable [4, 5]. Due to its high lipophilicity, diazepam readily crosses the placental barrier and may exert pharmacological effects on both maternal and fetal peripheral systems [6]. Although its central nervous system effects are well documented, its peripheral effects, particularly its vasodilatory and hypotensive properties [7, 8], may contribute to potential vascular and tissue‐specific impacts during gestation. To mitigate teratogenic risks, clinical guidelines emphasize monotherapy at the lowest effective dose and shortest duration, particularly avoiding exposure in the first‐trimester and polypharmacy [6].

Hypertensive disorders during pregnancy, including preeclampsia and eclampsia, lead to complications in approximately 5%–10% of pregnancies and remain a major cause of maternal and perinatal morbidity and mortality worldwide [9, 10]. There is consistent evidence that plasma homocysteine (Hcy) levels are increased two to threefold in women with preeclampsia compared to normotensive pregnant women, particularly in the third trimester, with concentrations increasing in parallel with disease severity [11–14]. These findings support the role of Hcy as a predictive marker for pregnancy‐induced hypertension [14, 15]. Hcy promotes vascular dysfunction through protein homocysteinylation, excessive generation of reactive oxygen species (ROS), and reduced nitric oxide bioavailability, leading to endothelial injury and impaired vasodilation [16, 17]. In parallel, hyperhomocysteinemia stimulates smooth muscle proliferation and inflammatory signaling, contributing to vascular remodeling and increased arterial stiffness [18–20]. These mechanisms support the use of Hcy‐treated human umbilical artery (HUA) as an experimental model that mimics key pathophysiological features of preeclampsia.

The human umbilical cord plays a key role in maternal–fetal exchange and serves as a valuable ex vivo model for the study of prenatal exposure and drug‐induced peripheral effects [21]. The high‐resistance flow pattern of HUAs makes them a common indicator of placental dysfunction in models of hypertensive disorders, such as hyperhomocysteinemia [22, 23]. In vitro models of hyperhomocysteinemia typically involve incubation of isolated blood vessels with elevated concentrations of Hcy ranging from 100 *μ*M to 1 mM, mimicking the pathophysiological conditions of preeclampsia [23–26].

Although most existing data on placental and pregnancy‐related vascular dysfunction come from human umbilical vein endothelial cell (HUVEC) models, HUAs offer several key advantages, as they preserve the intact vascular wall architecture, including both endothelium and smooth muscle, and enable direct assessment of vascular reactivity, oxidative injury, and structural alterations under well‐defined experimental conditions.

Despite the widespread use of diazepam in obstetrics, its effects on oxidative status, vascular morphology and functional reactivity within the umbilical circulation remain poorly characterized. Oxidative stress, defined as an imbalance between the generation of ROS and antioxidant defenses, is increasingly recognized as a central mediator of drug‐induced cellular damage [27]. Biochemical markers such as lipid peroxidation and changes in antioxidant enzyme activity provide valuable insights into oxidative alterations within HUA tissue following pharmacological exposure [28]. Simultaneously, histological assessment and TUNEL (terminal deoxynucleotidyl transferase dUTP nick end labeling) analysis of HUA can reveal structural disruptions, apoptotic activity, and potential impairments in vascular integrity [29]. Moreover, studying the vascular reactivity of HUAs in a hyperhomocysteinemic environment may provide new insights into the vasodilatory properties of diazepam and their potential therapeutic implications in hypertensive pregnancy. Therefore, the aim of this study was to investigate the effects of diazepam on oxidative stress parameters, histological changes, cell death–associated DNA fragmentation and vascular reactivity in HUAs under conditions of experimentally induced hyperhomocysteinemia.

## 2. Methods

### 2.1. Ethics Statement

The study protocol was reviewed and approved by the Ethics Committee of the University Clinical Center of the Republic of Srpska, Banja Luka, Republic of Srpska, Bosnia and Herzegovina (Approval No. 01–6211‐2724). Written informed consent was obtained from all 10 participants following a full explanation of the purpose, nature, and risks of the procedures, with patient anonymity strictly maintained.

### 2.2. Tissue Isolation and Experimental Design

HUA segments (2–3 mm) were isolated from umbilical cords collected postcaesarean section from healthy term pregnancies (≥ 38 weeks) at the Clinic for Gynecology, University Clinical Center Banja Luka (Republic of Srpska, Bosnia and Herzegovina). No pharmacological treatment was administered to the mothers prior to delivery.

**Figure 1:**
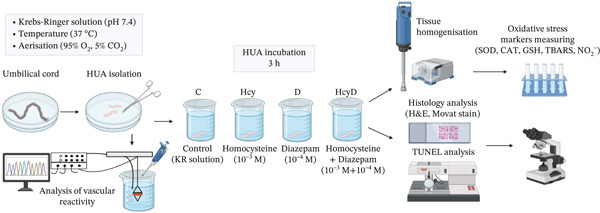
Illustration of the applied methodology using HUA segments for measuring vascular reactivity, oxidative stress markers, histological, and TUNEL analysis. Created with BioRender.

The isolated HUA segments were rinsed with ice‐cold saline, sectioned, and incubated in Krebs‐bicarbonate solution at 37°C for 3 h under continuous aeration with a gas mixture of 95% O_2_ and 5% CO_2_. To assess the effects of diazepam, hyperhomocysteinemia, and their combination on oxidative stress and vascular morphology, samples were divided into four treatment groups: C (control) group—HUA segments treated with Krebs‐bicarbonate solution only; D (diazepam) group—HUA segments treated with 100 *μ*mol/L diazepam in the buffer; Hcy group—HUA segments treated with 1 mM/L Hcy in the buffer; HcyD (combination) group—HUA segments treated with both 1 mM/L Hcy and 100 *μ*mol/L diazepam in the buffer (Figure 1). Supraphysiological concentrations of Hcy (1 mM) and diazepam (100 *μ*M) were selected based on prior in vitro vascular studies demonstrating reproducible mechanistic effects within these ranges, whereas lower concentrations produced minimal or inconsistent effects [30–32]. Following incubation, tissue samples were frozen and stored at −20°C until further analysis.

### 2.3. Tissue Homogenization

Frozen HUA samples were homogenized in ice‐cold phosphate buffer (pH 7.4) using an HG‐15D homogenizer (Witeg Labortechnik GmbH, Wertheim, Germany). The homogenates were centrifuged at 1200 × g for 10 min at 4°C. The resulting supernatants were collected for biochemical analysis.

### 2.4. Oxidative Stress Markers

Oxidative stress was assessed by quantifying pro‐oxidative markers: thiobarbituric acid reactive substances (TBARSs) and nitrite (NO_2_^−^), along with antioxidant markers including superoxide dismutase (SOD), catalase (CAT), and glutathione (GSH) in HUA homogenates. Lipid peroxidation was determined using the TBARS assay, involving reaction with 1% thiobarbituric acid (TBA) in the presence of 0.05 mol/L sodium hydroxide (NaOH), with absorbance measured at 530 nm [33]. NO_2_^−^ levels were measured using the Griess reagent [34], and SOD, CAT, and reduced GSH levels were quantified spectrophotometrically according to the protocols of Beutler [35, 36].

### 2.5. Histological Analysis

To examine structural changes in arterial morphology, HUA segments were fixed in 4% formaldehyde for 48 h. Samples were processed using the Leica TP 1020 automatic tissue processor (Leica Microsystems, Germany), embedded in paraffin using the Leica HistoCore Arcadia H system, and sectioned transversely at 4 *μ*m thickness with a Rotary 3003 microtome (pfmmedical, Germany). Sections were mounted on standard glass slides (Epredia, the Netherlands).

Histological staining included standard hematoxylin–eosin (H&E) and Movat pentachrome staining (ab245884, Abcam). For Movat staining, sections were rehydrated and stained with an elastic fiber solution, differentiated with 2% ferric chloride, incubated with iodine, rinsed, treated with 2% acetic acid, and stained with alcian blue for 25 min. This staining method differentiates connective tissue components such as collagen, elastin, muscle fibers, and fibrin.

Microscopic analysis was conducted using a Leica DM6000 microscope equipped with a Leica DFC310FX digital camera (Leica Microsystems, Germany).

The structural alterations were assessed using a semiquantitative damage scoring system, adapted from validated vascular pathology grading methods, previously described by Hernández‐Rodríguez et al. [37]. Briefly, the semiquantitative histological assessment was based on the severity and distribution of vascular lesions such as endothelial disruption, edema, fragmentation of smooth muscle cells, disruption of elastic fibers, and the presence of inflammatory infiltrates within the arterial wall, the extent of circumferential involvement, intimal hyperplasia, and the presence of granulomatous inflammation and multinucleated giant cells. Score 1 corresponded to normal histological architecture. Score 2 indicated mild, focal alterations with limited inflammatory and structural changes. Score 3 indicated moderate lesions involving a substantial portion of the vessel wall. Score 4 indicated severe, diffuse, and transmural injury associated with extensive inflammatory infiltrates and marked structural damage.

Characteristic morphological features of vascular injury were systematically evaluated, including endothelial disruption, edema, fragmentation of smooth muscle cells, disruption of elastic fibers, and the presence of inflammatory infiltrates. The analysis was conducted on all fields of view at magnifications of ×20 and ×40 on sections stained with H&E and Movat pentachrome. Each field of view at ×40 magnification was evaluated individually and assigned the appropriate semiquantitative score. The evaluator was blinded to the experimental groups, ensuring unbiased assessment.

### 2.6. TUNEL Analysis

Apoptotic cell death in HUA tissue was assessed using the TUNEL assay, which detects DNA fragmentation, a hallmark of apoptosis. The assay was performed on paraffin‐embedded HUA sections using a commercial TUNEL in situ kit (Elabscience, China; catalogue number: E‐CK‐A331), following the manufacturer′s instructions. After treatment with proteinase K for antigen retrieval and labeling with terminal deoxynucleotidyl transferase and fluorescein‐dUTP, nuclei were counterstained with DAPI. Slides were examined under a fluorescence microscope (Leica DM6000, Leica Microsystems, Germany), and TUNEL‐positive cells were quantified in multiple randomly selected fields. Results were expressed as the percentage of TUNEL‐positive nuclei relative to the total number of nuclei. This analysis allowed assessment of cell death–associated DNA fragmentation in the HUA vascular wall for four experimental groups.

### 2.7. Analysis of Vascular Reactivity

Vascular reactivity of HUA rings was evaluated as previously described [26]. Briefly, HUA rings were suspended in isolated organ baths containing 10 mL of Krebs‐bicarbonate solution, continuously aerated with 95% O_2_ and 5% CO_2_ at 37°C (pH 7.3–7.4). One end of each ring was attached to a fixed hook and the other to an isometric force transducer (MDE, SN: 20 K0910). Tension was recorded using an amplifier (MDE, SN: 20 K0912) and data acquisition system (IsoSys, MDE Research, Budapest, Hungary).

The rings were equilibrated for 60 min under unstretched conditions, with the solution replaced every 15 min. During equilibration, passive tension was gradually adjusted to 2.0 g. Before initiating experimental measurements, a reference contraction was elicited using potassium chloride (KCl, 40 mmol/L). Endothelial function in HUA was not routinely assessed with acetylcholine due to the marked variability in acetylcholine‐induced relaxation previously demonstrated in this model [26].

To assess the effects of treatments, test compounds were added to the organ bath 60 min before cumulative concentration–response curves to serotonin (0.001–100 *μ*mol/L) were generated. Diazepam (100 *μ*mol/L), Hcy (1 mmol/L), or their combination (100 *μ*mol/L diazepam + 1 mmol/L Hcy) were tested. Results are expressed as a percentage of the maximal KCl‐induced contraction.

### 2.8. Statistical Analysis

Statistical analysis was performed using Student′s paired *t*‐test (SigmaPlot v.11, Systat Software), with *p* < 0.05 considered statistically significant. Data normality was assessed using the Shapiro–Wilk test prior to analysis. The paired design was applied because arterial rings obtained from the same donors were exposed to different experimental conditions, allowing each measurement to be directly compared with its corresponding within‐donor control. In each prespecified group comparison, oxidative stress markers, histological findings, and cell death–associated DNA fragmentation were compared with the corresponding control group (C or HcyD), whereas vascular reactivity was compared with the serotonin‐alone condition. Results are presented as the mean ± SD of *n* replicates, where *n* represents the number of HUA rings tested per protocol, each obtained from a different umbilical cord. The analysis was restricted to a limited number of a priori, biologically driven pairwise comparisons rather than a full repeated‐measures framework. Given the small sample size (*n* = 6), more complex approaches such as repeated‐measures ANOVA or mixed‐effects models were not applied due to the difficulty in reliably verifying their assumptions and the risk of unstable estimates. Graphs were created with GraphPad Prism Version 8.

## 3. Results

### 3.1. Effects of Diazepam and Hyperhomocysteinemia on Oxidative Stress Markers in a HUA

**Figure 2:**
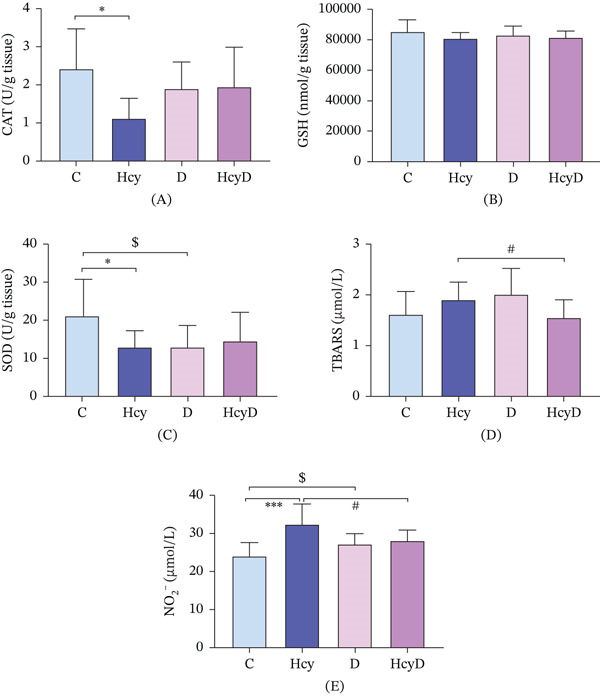
Effects of diazepam and hyperhomocysteinemia on oxidative stress markers in HUA tissue. (A) CAT activity (*n* = 8), (B) reduced GSH concentration (*n* = 10), (C) SOD activity (*n* = 10), (D) TBARS levels (*n* = 10), and (E) NO_2_^−^ levels (*n* = 9) in HUA homogenates from control (C), homocysteine‐treated (Hcy), diazepam‐treated (D), and combined homocysteine + diazepam (HcyD) groups. Data are presented as mean ± SD (n = number of HUA rings from individual cords).  ^∗^ *p* < 0.05,  ^∗∗∗^ *p* < 0.001 vs. control group; ^#^ *p* < 0.05 vs. homocysteine + diazepam (HcyD) group; ^$^ *p* < 0.05 diazepam‐treated (D) vs. control group.

Hyperhomocysteinemia, modeled by in vitro exposure to high level of Hcy (Hcy group), resulted in a significant reduction in the activities of key antioxidant enzymes, CAT (2.43 U/g tissue, 95% CI 1.47–3.39; Hcy: 1.12 U/g tissue, 95% CI 0.58–1.67; *p* = 0.004, Figure 2A) and SOD (C: 21.25 U/g tissue, 95% CI 13.95–28.55; Hcy: 13.02 U/g tissue, 95% CI 10.02–16.03; *p* = 0.023, Figure 2C). The pro‐oxidative effect was accompanied by a significant increase in NO_2_^−^ levels, compared to the control group (C: 24.1 *μ*mol/L, 95% CI 21.4–26.8; Hcy: 32.5 *μ*mol/L, 95% CI 28.7–36.3; *p* < 0.001; Figure 2E). Although TBARS levels (Figure 2D) and reduced GSH concentrations (Figure 2B) showed trends towards increased oxidative stress, these changes were not statistically significant.

Treatment with diazepam alone (Group D) resulted in reduced SOD activity (Figure 2A) and NO_2_^−^ levels (Figure 2E) compared to control group, suggesting a pro‐oxidative effect. This trend was also observed in CAT activity (Figure 2C) and TBARS levels (Figure 2D), although these changes did not reach statistical significance. Interestingly, diazepam exhibited a dual effect: Although it impaired some antioxidant defenses, it significantly reduced NO_2_^−^ levels compared to the Hcy group (Hcy: 32.5 *μ*mol/L, 95% CI 28.7–36.3; D: 27.33 *μ*mol/L, 95% CI 25.5–29.2; *p* = 0.012; Figure 2E), indicating a potential nitrosative stress‐modulating property. No significant change in GSH levels was observed following diazepam treatment (Figure 2B).

Cotreatment with diazepam in the hyperhomocysteinemia group (HcyD) significantly reduced TBARS (Hcy: 1.91 *μ*mol/L, 95% CI 1.65–2.18; HcyD: 1.57 *μ*mol/L, 95% CI 1.31–1.82; *p* = 0.044; Figure 2D) and NO_2_^−^ levels (Hcy: 32.49 *μ*mol/L, 95% CI 28.73–36.25; HcyD: 28.14 *μ*mol/L, 95% CI 26.19–30.09; *p* = 0.032; Figure 2E) compared to the Hcy group, suggesting a partial restoration of redox balance. However, the activities of CAT, SOD, and GSH remained unchanged compared to the Hcy group (Figure 2A–C). These findings indicate that although diazepam alone may impair certain antioxidant mechanisms, its coadministration under hyperhomocysteinemic conditions may confer partial antioxidant protection by attenuating lipid peroxidation and nitrosative stress.

### 3.2. Histological Evaluation of HUA Wall Integrity

**Figure 3:**
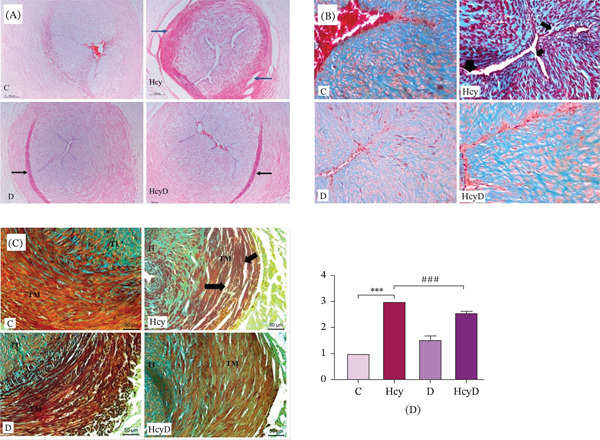
Representative microphotographs of HUA cross sections from control (C), homocysteine (Hcy)‐treated, diazepam (D)‐treated, and combined homocysteine + diazepam (HcyD) groups. (A) H&E staining (10x, scale bar = 100 *μ*m). C shows normal histology; D shows near‐normal structure with increased extracellular matrix; Hcy shows reduced wall thickness, irregular lumen, and smooth muscle delamination (blue arrows); HcyD shows local smooth muscle delamination and increased intimal extracellular matrix (the artifact is marked with a thin black arrow). (B) Movat pentachrome staining (40x, scale bar = 100 *μ*m). C and D show intact endothelium and normal subendothelial/smooth muscle layers; Hcy shows endothelial damage (thick black arrows) and condensed subendothelial layer; HcyD shows preserved endothelium with loosened subendothelial layer (unstained spaces). (C) Movat pentachrome staining (20x, scale bar = 50 *μ*m). C, D, and HcyD show normal tunica intima/media; Hcy displays subendothelial damage, disrupted media (unstained spaces), contracted smooth muscle, and cell delamination (arrows). Minor differences in color shade between panels reflect higher illumination/brightness settings required at greater magnification and do not indicate differences in the staining protocol. (D) Vascular damage scores (mean ± SD, *n* = 3, 10 fields per HUA ring). All treatments increased damage vs. C; D had less damage than Hcy and HcyD; HcyD showed reduced damage vs. Hcy.  ^∗∗∗^ *p* < 0.001; ^###^ *p* < 0.001 (between indicated groups).

The structural effects of diazepam and hyperhomocysteinemia on the HUA wall were assessed by H&E staining (Figure 3A), Movat pentachrome staining of the endothelium (40x, Figure 3B) and smooth muscle (20x, Figure 3C), and semiquantitative scoring (Figure 3D).

In the control (C) group, H&E staining revealed preserved vascular architecture, characterized by an intact endothelial monolayer in the tunica intima, a well‐organized subendothelial connective tissue, and a tunica media composed of compact smooth muscle bundles interspersed with loose connective tissue (Figure 3A [C]). Movat pentachrome staining confirmed structural integrity, with a continuous endothelium overlying ground substance‐rich subendothelial connective tissue (blue), collagen fibers (yellow‐green), and sparse elastic fibers (black), with organized smooth muscle bundles (orange‐red) in the tunica media (Figure 3B [C],C [C]).

The hyperhomocysteinemia (Hcy) group displayed pronounced histological damage, including thinning of the vessel wall, irregular lumen morphology, and delamination of smooth muscle cells in the tunica media (Figure 3A [Hcy]). Movat staining further highlighted these changes, showing discontinuity of the endothelial layer, reduced ground substance in the intima (Figure 3B [Hcy]), disorganized smooth muscle architecture, and marked loss of medial connective tissue (Figure 3C [Hcy]).

In the diazepam‐treated group, H&E staining showed largely preserved arterial structure, with intact endothelium and mildly expanded subendothelial connective tissue (Figure 3A [D]). Movat staining revealed increased extracellular fluid in the subendothelial layer, indicated by widened uncoloured spaces between collagen fibers and a reduction in endomysial connective tissue (Figure 3B [D],C [D]).

Cotreatment with diazepam in the HcyD group resulted in attenuated histological damage. H&E sections showed only localized smooth muscle delamination and endothelial disruption, along with increased extracellular matrix deposition and subendothelial hyperplasia in the intima (Figure 3A [HcyD]). Movat staining confirmed these findings, showing increased accumulation of subendothelial connective tissue and relatively well‐preserved smooth muscle organization, despite focal areas of medial disarray and reduced endomysium (Figure 3B [HcyD],C [HcyD]).

All treatment groups (Hcy, D, and HcyD) showed a significant increase in damage compared to control (*p* < 0.001). However, damage in the D group was significantly lower than in the Hcy and HcyD groups, and HcyD showed a significant reduction in damage compared to Hcy alone (*p* < 0.001, Figure 3D).

### 3.3. TUNEL Analysis in HUA Tissue

**Figure 4:**
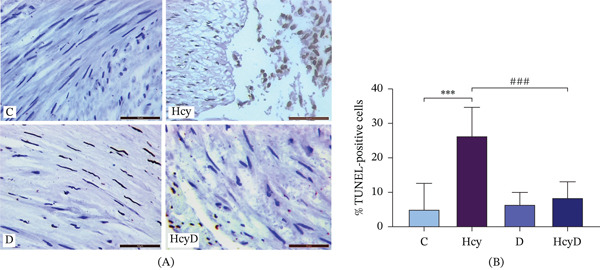
TUNEL assay for detection of cells with DNA fragmentation in HUA tissue sections, magnifcation 400x. (A) The control group showed minimal TUNEL‐positive staining. The Hcy group exhibited a significant increase in DNA fragmentation, indicated by intense brown nuclear staining. The D group showed a similar pattern to the control, with few TUNEL‐positive cells. Cotreatment with diazepam (HcyD) markedly reduced Hcy‐induced apoptosis. (B) Quantitative analysis of TUNEL‐positive cells (mean ± SD, *n* = 3, 10 fields per HUA ring).  ^∗∗∗^ *p* < 0.001, ^###^ *p* < 0.001 (between indicated groups).

TUNEL analysis was used to evaluate the effects of hyperhomocysteinemia and diazepam on cell death–associated DNA fragmentation in HUA tissue. Exposure to a high concentration of Hcy (1 mM) significantly increased the number of TUNEL‐positive smooth muscle and endothelial cells, indicating enhanced apoptotic activity (*p* < 0.001). In contrast, cotreatment with diazepam markedly reduced the apoptotic index in the hyperhomocysteinemia group, suggesting a protective effect. Diazepam alone showed a similar pattern and distribution of TUNEL‐positive cells as the control group, with only a few positively stained smooth muscle cells observed, as shown by the characteristic brown nuclear staining (Figure 4A). Quantitative analysis revealed minimal TUNEL‐positive staining in the control group, a significant increase in DNA fragmentation in the Hcy group, and a pattern similar to control in the D group. Cotreatment with diazepam (HcyD) significantly attenuated Hcy‐induced apoptosis (*p* < 0.001, Figure 4B). Although TUNEL‐positive nuclei were predominantly observed in the intimal and medial layers, the lack of cell‐type–specific colabeling and the inability of TUNEL to distinguish apoptosis from other forms of cell death limit precise characterization of the affected cell populations and death pathways.

### 3.4. Vascular Reactivity of HUA Rings

**Figure 5:**
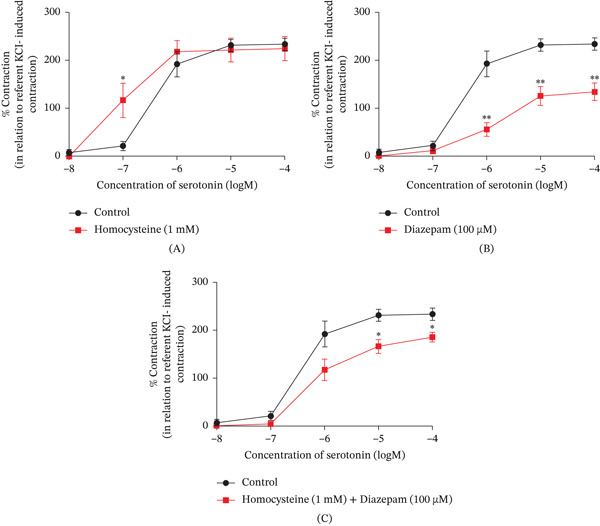
Effects of diazepam and hyperhomocysteinemia on serotonin‐induced contraction in isolated HUA rings. Concentration–response curves for serotonin (logM) were generated in control rings (black circles, *n* = 6) and rings (red squares) preincubated for 60 min with (A) 1 mM homocysteine (*n* = 6), (B) 100 *μ*M diazepam (*n* = 6), and (C) 1 mM homocystein + 100 *μ*M diazepam (*n* = 6). Results (mean ± SD) are expressed as percentage of contraction in relation to reference KCl‐induced contraction. Parentheses indicate the number of HUA preparations studied.  ^∗^ *p* < 0.05,  ^∗∗^ *p* < 0.01, significantly different E_max_ values from the respective control.

One‐hour incubation of HUA rings with 1 mM Hcy resulted in a significant leftward shift of the serotonin concentration–response curve, indicating increased vascular sensitivity to serotonin (*p* = 0.028; Figure 5A). Incubation with 100 *μ*M diazepam produced a significant downward shift in the serotonin concentration–response curve, demonstrating reduced contractile responsiveness of HUAs to serotonin (*p* = 0.001; Figure 5B). However, when diazepam was applied following Hcy incubation, its inhibitory effect on serotonin‐induced contraction was attenuated. Although the downward shift was less pronounced, it remained significant compared to the control group (*p* = 0.015, Figure 5C).

## 4. Discussion

This study provides the first evidence for the influence of diazepam on oxidative stress, histopathological changes, cell death–associated DNA fragmentation, and vascular function in HUA tissue in an in vitro model of hyperhomocysteinemia.

Methionine, an essential amino acid for protein synthesis, also functions as an important methyl group donor via its conversion to S‐adenosylmethionine (SAM). After SAM‐dependent transmethylation, S‐adenosylhomocysteine (SAH) is formed and hydrolyzed to Hcy and adenosine. Hcy can be remethylated back to methionine or metabolized via the transsulfuration pathway to cystathionine and subsequently to cysteine and *α*‐ketobutyrate. Disruptions in transmethylation and transsulfuration during pregnancy have been associated with unfavorable outcomes, including neural tube defects, pre‐eclampsia, miscarriage, and preterm birth [38]. The toxicity of Hcy is attributed to its covalent binding to proteins, a process known as homocysteinylation, which alters protein function and increases proportionally with elevated plasma Hcy levels [16, 17]. Hyperhomocysteinemia, which is recognized as a predictor of pregnancy‐induced hypertension [14, 15], contributes to vascular dysfunction via multiple mechanisms, including oxidative stress, endothelial damage, smooth muscle proliferation, and inflammation [18–20, 28, 39]. Although some biomarkers of oxidative stress in umbilical cord blood, such as CAT and GSH, have been linked to fetal growth restriction and proposed as potential diagnostic markers [40], similar analyses of oxidative stress markers in HUA tissue have not yet been reported.

In our study, in vitro hyperhomocysteinemia (Hcy, 1 mM) was associated with increased oxidative stress in HUA tissue, as evidenced by a significant decrease in CAT and SOD activities, along with a marked increase in NO_2_^−^ concentrations. These findings are consistent with those of Feng et al. [41], who showed that hyperhomocysteinemia induced oxidative stress in HUVECs characterized by increased levels of malonyl dialdehyde (MDA) and decreased levels of SOD and endothelial nitric oxide synthase (eNOS). Interestingly, diazepam alone (100 *μ*M) exerted a dual effect on oxidative status by significantly reducing SOD activity, which suggests a pro‐oxidative effect, while also lowering NO_2_^−^ levels, pointing to a potential modulatory role in nitrosative stress. In the presence of hyperhomocysteinemia, cotreatment with diazepam resulted in a significant decrease in MDA and NO_2_^−^ levels, without altering the activities of CAT, SOD, or GSH. Numerous studies have investigated the role of ROS‐mediated pro‐oxidative mechanisms in the brain after diazepam administration. Musavi and Kakkar [42, 43] reported that even a single dose of diazepam can induce region‐specific and modulatory oxidative changes in nervous tissue. In contrast, other studies have shown that diazepam exerts antioxidant and protective effects in models of acute stress [44] and ethanol‐induced oxidative brain damage and neurodegeneration [45]. Similarly, its effects on oxidative stress in peripheral tissues appear to be tissue‐specific. For example, El‐Sokkary [46] observed increased TBARS levels in rat liver with decresed GSH content and SOD activity, whereas Jiang et al. [47] found no significant changes in SOD, GSH, MDE, or NO levels in rat cardiac tissue. Although the previous results were from long‐term in vivo diazepam treatment, our study examined short‐term exposure in HUA tissue, revealing that despite the potential of diazepam to impair certain antioxidant defenses, its coadministration with high concentration of Hcy may provide selective antioxidant benefits by reducing lipid peroxidation and nitrosative stress. Our findings are in align with those of Jiang et al. [47], who reported that diazepam treatment significantly reduced cardiac oxido‐nitrosative stress induced by ischemia‐reperfusion, with even greater efficacy than diazepam in controls. However, the observed reduction in NO_2_^−^ may reflect both decreased NO synthesis and/or increased NO scavenging under the present experimental conditions, although the available data do not allow a definitive distinction between these mechanisms.

HUAs are considered atypical blood vessels that lack adventitia and innervation and are instead surrounded by Wharton′s jelly. Histological analysis showed that hyperhomocysteinemia leads to significant thinning of vessel walls, destruction of the endothelium, smooth muscle delamination, and loss of medial connective tissue. Although there are no publications reporting the effects of hyperhomocysteinemia in HUAs, other vascular studies strongly suggests that hyperhomocysteinemia contributes to endothelial damage and dysfunction, smooth muscle cell proliferation or delamination, matrix remodeling with collagen/elastin deposition, intimal hyperplasia and medial fibrosis [48, 49]. Diazepam alone preserved arterial integrity, whereas cotreatment attenuated hyperhomocysteinemia‐induced damage, resulting in focal endothelial and smooth muscle alterations, an increase in subendothelial matrix, and largely preserved medial architecture. These findings provide new insights into the effects of diazepam on vascular histology, in contrast to existing data from high‐dose animal models or cell cultures, which typically reflect chronic exposure and may not apply to short‐term clinical use. Studies in animal models using high‐dose diazepam (~5 mg/kg) have reported thickened organ capsules and cellular disorganization in the liver, kidney, spleen, and thymus, along with enlarged interstitial spaces in renal and pulmonary tissues, and abnormal cardiac intercalated discs [50]. These findings are consistent with our observation of subendothelial connective tissue expansion and increased extracellular fluid accumulation in HUA sections.

The literature shows that diazepam has both pro‐ and antiapoptotic effects, with fewer studies reporting proapoptotic activity in immune [51] and neural cells [52], and more evidence supporting its anti‐apoptotic and neuroprotective roles, particularly in rat hippocampal neurons after cerebral ischemia [53–55], in human neutrophils [56], in myocardial ischemia‐reperfusion injury in rats [47], and in the lipopolysaccharide‐induced inflammatory model in mice [57].

Our findings confirmed the protective effect of diazepam, as cotreatment significantly reduced the proportion of TUNEL‐positive cells in the hyperhomocysteinemia group. However, the efficacy of diazepam seems to depend on the dose, duration of exposure, and tissue type, which should be taken into account when assessing clinical relevance. Future work combining TUNEL with complementary markers (e.g., cleaved caspase‐3 and BAX/Bcl‐2) and cell‐type–specific labeling will be necessary to more precisely define the nature and cellular origin of vascular injury in this model.

Vascular reactivity testing showed that hyperhomocysteinemia increased HUA contractile responses to serotonin, although diazepam alone reduced these contractions, confirming its vasodilatory effect. Previous study reported that diazepam induces over 50% vasodilation in serotonin‐precontracted HUAs, likely by modulating voltage‐ and receptor‐operated Ca^2+^ influx via the 18 kDa translocator protein (TSPO) through an endothelium‐independent mechanism [58]. Additionally, earlier studies have shown that 300 *μ*M Hcy causes a marked leftward shift in the serotonin concentration‐response curve [26]. Using a slightly higher Hcy concentration, consistent with other in vitro hyperhomocysteinemia models [24, 25], we observed a similar potentiation of serotonin‐induced contractility. However, cotreatment with diazepam and Hcy attenuated the inhibitory effect of diazepam, suggesting an interaction in modulating HUA reactivity. These results suggest that the interplay between diazepam and hyperhomocysteinemia depends on both dose and duration of exposure. Considering the 1‐h incubation protocol used in this study, it is reasonable to assume that diazepam′s effect on reducing contractile responsiveness to serotonin might be diminished under prolonged hyperhomocysteinemic conditions. Although hyperhomocysteinemia is known to impair vascular function through multiple pathways, the specific mechanisms responsible for the attenuation of diazepam‐induced vascular effects in this setting remain undefined and will require targeted mechanistic studies in future work.

The main limitation of this study is the use of diazepam and Hcy concentrations exceeding pharmacological and physiological ranges, which reduces the translational applicability of the results. Lokeshwari et al. [59] reported that patients with preeclampsia exhibit a mean Hcy concentration of approximately 30 *μ*mol/L, about 33 times lower than the concentration used in our in vitro model. Similarly, Park et al. [30] found that supraclinical diazepam levels reach around 5 *μ*mol/L, 20 times lower than those applied in our incubation experiments. The use of high concentrations in isolated tissues is common, reflecting the lower sensitivity of these models and the specific conditions of in vitro studies, including the absence of pharmacokinetic processes, immune responses, and inter‐tissue interactions. Although the concentrations used exceed clinically observed levels, they were selected to overcome the limited responsiveness of isolated vascular tissue and to enable detection of pharmacodynamic interactions under controlled in vitro conditions. Accordingly, the findings should be interpreted as mechanistic insights rather than quantitative predictors of in vivo effects. The second limitation is the exploratory nature of the study and its statistical approach. A small sample (*n* = 6) was analyzed using prespecified paired *t*‐tests for hypothesis‐driven comparisons rather than a full factorial design. Although multiple comparisons were not formally adjusted, they were defined a priori, and strict multiplicity correction could have increased the risk of Type II error. Accordingly, the findings should be interpreted with caution and considered hypothesis‐generating. The third limitation is that the mechanisms underlying the protective effects of diazepam remain unclear. Although diazepam primarily acts as a positive allosteric modulator of GABA_A_ receptors, its vascular effects may also involve interactions with other molecular targets, including ion channels and membrane‐associated proteins. The contribution of these pathways, together with the expression and functional role of GABA_A_ receptor subtypes, was not investigated in the present study. Diazepam is also a highly lipophilic molecule that readily enters cells and can bind to TSPO located in the outer mitochondrial membrane, a protein implicated in the regulation of mitochondrial function, oxidative stress, and apoptosis. Consequently, modulation of mitochondrial ROS generation through TSPO may contribute to the attenuation of Hcy‐induced vascular injury. This possibility is supported by the observed reduction in oxidative stress marker TBARS together with the partial restoration of vascular function in HcyD group, findings that are consistent with improved redox homeostasis and preservation of NO signaling. However, because NO bioavailability, cGMP signaling, and the involvement of TSPO and GABA_A_ receptors were not directly investigated, these mechanisms remain hypothetical. Further studies using selective pharmacological ligands together with direct assessment of receptor expression, mitochondrial function, ROS production, and NO signaling are required to clarify the molecular basis of the vascular protective effects of diazepam. Another limitation is the lack of endothelial‐specific functional assays, as endothelial function in HUA was not routinely assessed. In the HUA preparation, acetylcholine‐induced relaxation has been described as highly variable and often modest or absent, which reduces the robustness and reproducibility of endothelial function testing and complicates the interpretation of negative responses [26]. Consequently, our conclusions regarding endothelial dysfunction are based primarily on histological evidence of endothelial disruption.

## 5. Conclusion

Our findings confirm that hyperhomocysteinemia exerts pro‐oxidative, proapoptotic, and procontractile effects on HUA, accompanied by pronounced histological damage. At the same time, diazepam showed pleiotropic actions on HUA, modulating oxidative stress markers, histological features, and vascular tone. Under hyperhomocysteinemia conditions, diazepam cotreatment afforded partial protection, consistent with reduced lipid peroxidation and nitrosative stress, less histological injury, and a lower proportion of TUNEL‐positive cells. These results suggest that diazepam can partially attenuate hyperhomocysteinemia‐induced vascular alterations in an in vitro model that mimics certain pathophysiological features of preeclampsia, although any clinical implications remain highly speculative given the supraphysiological concentrations used.

## Author Contributions

**Milica Gajić Bojić:** writing – original draft, investigation, formal analysis. **Zvjezdana Ritan Mićić:** writing – original draft, visualization, software. **Aneta Stojmenovski:** investigation. **Anđela Bojanić:** investigation. **Sanja Jovičić:** formal analysis, writing – review and editing. **Milka Matičić:** investigation. **Nebojša Mandić-Kovačević:** investigation. **Snežana Uletilović:** investigation. **Ljiljana Amidžić:** investigation. **Miroslav Savić:** writing – review and editing, project administration, methodology, supervision. **Ranko Škrbić:** writing – review and editing, supervision, resources.

## Funding

No funding was received for this manuscript.

## Conflicts of Interest

The authors declare no conflicts of interest.

## Data Availability

The datasets generated and analyzed during the current study are available in the Zenodo repository at 10.5281/https://doi.org/10.5281/zenodo.16808767
